# Cybersecurity is imperative in robotic arthroplasty

**DOI:** 10.1051/sicotj/2026019

**Published:** 2026-05-05

**Authors:** Vaibhav Bagaria, Raju Vaishya, Abhishek Vaish, Sébastien Lustig

**Affiliations:** 1 Sir HN Reliance Foundation Hospital Mumbai India; 2 Department of Orthopaedics and Joint Replacement Surgery, Indraprastha Apollo Hospitals Sarita Vihar, New Delhi 110076 India; 3 Hôpital de la Croix Rousse, Université Claude Bernard Lyon I Lyon France

**Keywords:** Robotic arthroplasty, Cybersecurity, Robotic-assisted surgery, Medical device security, Patient safety

## Abstract

Robotic platforms have revolutionized arthroplasty through precision and patient-specific planning, yet introduce cyber-physical vulnerabilities in interconnected surgical ecosystems. Recent incidents, including the 2026 cyber-attack, highlight operational risks despite low direct intraoperative threats. Proactive cybersecurity, via FDA-aligned secure design, institutional audits, and surgeon vigilance, is imperative to safeguard patient safety and trust in precision orthopedics.

Over the past decade, robotic platforms have redefined joint replacement surgery. By enabling patient-specific planning, real-time feedback, and constrained execution, these systems have enhanced implant positioning and reproducibility. Robotics has shifted the paradigm from conventional alignment techniques toward individualized arthroplasty, contributing to improved functional outcomes and surgeon confidence [1]. However, these advances have also transformed the operating room (OR) into a digitally interconnected ecosystem [2]. Robotic systems depend on the seamless integration of imaging, planning software, navigation tools, and networked computing infrastructure. As such, they represent cyber-physical systems, where digital inputs directly influence physical surgical actions.

In several commercial sectors, such as aviation, energy, and finance, cybersecurity has long been recognized as an essential component of operational safety. Healthcare, however, has only recently begun to appreciate the implications of cyber vulnerabilities [3]. Hospitals worldwide have experienced ransomware attacks that shut down operating rooms, delayed patient care, compromised medical records, and imaging systems [4, 5].

Recently, a widely reported cyberattack on a leading orthopedic implant maker on March 11, 2026, disrupted its global internal Microsoft environment, leading to operational challenges in order processing, manufacturing, and shipping (https://www.stryker.com/us/en/about/news/2026/a-message-to-our-customers-03-2026.html). Fortunately, the incident resulted in no ransomware or malware and was contained to corporate IT systems. The company has affirmed that products, including their Robotic-Arm Assisted Surgery System, remain fully safe to use, as it is a non-connected device relying on encrypted, offline USB/flash drive planning with built-in validation checks (e.g., automated quality verification and error detection for mismatched plans). No compromise of surgical devices, intraoperative systems, or patient data occurred.

Globally, healthcare systems have increasingly faced ransomware attacks that disrupt hospital operations, delay procedures, and compromise access to clinical data [6, 7]. In parallel, experimental work has demonstrated the feasibility of manipulating medical imaging using adversarial techniques, raising concerns about the integrity of diagnostic and preoperative planning data [8]. While these risks remain largely theoretical in robotic arthroplasty, their implications are significant.

Importantly, contemporary robotic systems incorporate robust safety mechanisms. Surgeons remain in control, robotic execution is constrained within predefined boundaries, and intraoperative verification steps provide additional safeguards. The likelihood of a cyberattack directly altering surgical execution remains extremely low. Nonetheless, cybersecurity must be reframed as a core component of patient safety rather than a peripheral technical concern [9].

Potential vulnerabilities include unauthorized modification of preoperative planning datasets, disruption of calibration protocols, and system downtime resulting from network breaches [10]. Even transient interruptions can affect workflow in time-sensitive operative settings. These risks highlight the need for proactive, rather than reactive, cybersecurity strategies. A recent stakeholder analysis of robotic-assisted surgery cybersecurity highlights persistent gaps, including limited clinician awareness of organizational policies, insufficient inter-stakeholder data sharing, and vulnerabilities from external threats or supply-chain dependencies [11]. While direct intraoperative manipulation remains improbable due to constrained execution and safety boundaries, such findings reinforce the value of systematic risk assessments (e.g., via Failure Modes, Effects, and Criticality Analysis) to identify and prioritize failure modes in Robotic Assisted Surgery (RAS) ecosystems.

Mitigation requires a shared responsibility by the manufacturers, institutions, and surgeons [12, 13]:

- *Manufacturers* must continue to strengthen secure software design, encryption standards, and regulatory compliance. They must align with evolving FDA requirements, including the February 2026 update to “Cybersecurity in Medical Devices: Quality Management System Considerations and Content of Premarket Submissions,” which enforces secure-by-design principles, SBOMs, and post market vulnerability management for devices with cybersecurity risks (https://www.fda.gov/regulatory-information/search-fda-guidance-documents/cybersecurity-medical-devices-quality-management-system-considerations-and-content-premarket)*.* While many robotic arthroplasty platforms (including Mako) minimize exposure through air-gapped or limited-connectivity architectures, these standards underscore the need for ongoing risk assessments even in ostensibly offline systems. Open-source robotic architectures may offer an unexpected advantage: transparency. Systems that can be independently scrutinized by a global community of engineers and clinicians may ultimately prove more secure than closed systems whose vulnerabilities remain hidden [13].
- *Institutions* should implement robust network protections, conduct regular cybersecurity tabletop exercises specific to robotic platforms, verify offline planning integrity, and maintain contingency protocols for supply disruptions (as seen in the Stryker case).
- *Surgeons,* while not cybersecurity experts, must develop situational awareness regarding the digital integrity of the systems they rely upon, much as they ensure sterility and mechanical accuracy in the OR. Additionally, surgeons should routinely confirm plan-file integrity (e.g., via checksums or vendor validation) before loading and remain vigilant for any anomalous system behavior, treating digital verification as parallel to mechanical checks.

Orthopedics has consistently embraced innovation – from arthroscopy to navigation and robotics, each advancing patient care while introducing new responsibilities. As surgical environments evolve to incorporate artificial intelligence (AI), cloud computing, and real-time analytics, the convergence of medicine and digital infrastructure will deepen [14, 15]. The future of surgery will be shaped not only by technology, but by how well we balance technique, technology, and the inevitable transition between the two [16].

Orthopedic surgery has always evolved at the intersection of innovation and responsibility. Robotic arthroplasty represents a major milestone in precision surgery. Ensuring the security and resilience of these systems is essential to preserving both patient safety and professional trust. As the recent incident demonstrates, even non-connected surgical technologies exist within broader digital ecosystems; proactive cybersecurity fortifies not only devices but operational continuity. In the digital era, safeguarding the integrity of our machines and protecting the surgical technology from cyber threats may become as fundamental as maintaining sterility itself and as essential as mastering the scalpel.

## Data Availability

The data for this paper are available in the public domain.
